# Editorial: Frontiers in Chemistry-Rising Stars: Asia

**DOI:** 10.3389/fchem.2021.811459

**Published:** 2021-11-24

**Authors:** Lei Guo, Jyotirmayee Mohanty, Wukun Liu, Taner Yonar, Hongyan Sun, Tsuyoshi Minami, Jafar Soleymani, Basem Moosa, Qianxiong Zhou

**Affiliations:** ^1^ School of Material and Chemical Engineering, Tongren University, Tongren, China; ^2^ Radiation and Photochemistry Division, Bhabha Atomic Research Centre, Mumbai, India; ^3^ Jiangsu Collaborative Innovation Center of Chinese Medicinal Resources Industrialization, Nanjing University of Chinese Medicine, Nanjing, China; ^4^ Environmental Engineering Department, Engineering Faculty, Bursa Uludag University, Bursa, Turkey; ^5^ Department of Chemistry and Center of Super-Diamond and Advanced Films (COSDAF), City University of Hong Kong, Hong Kong SAR, China; ^6^ Institute of Industrial Science, The University of Tokyo, Tokyo, Japan; ^7^ Pharmaceutical Analysis Research Center, Tabriz University of Medical Sciences, Tabriz, Iran; ^8^ Physical Science and Engineering Division, King Abdullah University of Science and Technology, Thuwal, Saudi Arabia; ^9^ Key Laboratory of Photochemical Conversion and Optoelectronic Materials, Technical Institute of Physics and Chemistry, Chinese Academy of Sciences, Beijing, China

**Keywords:** organic chemistry, nanocomposites, chemical biology, sustainable chemistry, biomolecular marker, cytotoxicity, antifungal and antibacterial activities, biodegradable plastics

Rising Stars: Asia is dedicated to collecting the high-quality work of outstanding researchers in the early stages of their independent careers, working across the Asian continent including the countries like China, India, Saudi Arabia, South Korea, Jordan, and Pakistan in the field of chemistry. This collection aims to showcase research from all fields of chemistry, *i.e.*, nanoscience, inorganic chemistry, green sustainable chemistry, medicinal and pharmaceutical chemistry, analytical chemistry, computational chemistry and more prominently, the chemical biology. This collection also presents advances in theory, and methodology, along with applications to fascinating problems.

Chemical biology mainly deals with the application of chemical techniques/methods, analysis, and chemicals produced through synthetic chemistry, to the study and manipulation of biological processes. Chemicals including natural and unnatural small molecules and drugs are used to address biological problems at a mechanistic level. These studies are encouraging and have inspired outstanding Asian researchers to design new compounds and more advanced analytical techniques to maximize the chemical results of their research. The primary objective of this research topic is to explore the latest developments in the field, focusing on design and synthesis of novel compounds, and/or their computational optimization as well as their biological applications.

Bharti et al. successfully demonstrated the green synthesis of crystalline gold-zinc oxide nanocomposites under organic solvent-free conditions at room temperature. They have achieved stable Au-ZnO/Zn(OH)_2_ nanocomposites by varying the reaction time and reagent concentrations. The *in situ* embedded gold nanoparticles enhance the luminescent properties of the nanocomposite materials which have been explored as bioimaging materials in human cells and applied for visible light–induced photodegradation of rhodamine-B dye. The study can be utilized to design and develop luminescent nanocomposite materials for bioimaging and dye degradation applications.

Sharfalddin et al. synthesized five transition metal complexes of sulfaclozine with Cu(II), Co(II), Ni(II), Zn(II), and Fe(II), and characterized their structural properties through various measurements. The conclusions drawn by theoretical calculations are consistent with the experimental results, indicating that copper and nickel in the metal complex have good coordination stability. DNA binding titration and molecular docking simulation have been utilized to evaluate the bio-activities of the characterized complexes. These results were supported by *in vitro* cytotoxicity assays showing that the Cu(II) and Ni(II) complexes display promising antitumor activity against colon and breast cancer cell lines.

Chen et al. synthesized seventeen kinds of glycosides of 1,3,4-thiadiazole derivatives and studied their antifungal activities. According to the structure-activity relationship analysis, the type and position of the substituents of the benzene ring in the derivatives play an important role in antifungal activity. The *in vitro* antibacterial activity experiments show that the derivatives have good antibacterial activity when the para-substituent is a fluorine atom or a nitro group.

Tian et al. investigated the chemical, phytochemical, toxicological and pharmacological properties of *Vaccaria segetalis* in the past 40 years. They summarized a large number of literatures, and mainly introduced the research progress of *Vaccaria segetalis* in detail from the aspects of its composition, clinical application, and biological activity.

Malik et al. synthesized eight new pyran-linked phthalazinone pyrazole hybrid compounds and evaluated their cytotoxicity in lung cancer and cervical cancer cells, respectively. The results show that the hybrid compound of methyl substitution on pyrazole and two cyano groups on pyran has a good inhibitory response to both types of cells. In addition, molecular modeling studies have shown that the two kinds of hybrid compounds have higher binding affinity to proteins and show excellent drug likelihood.

Zhu et al. introduced the development of isocyanide in biomolecular labeling from three strategies: two-component bio-orthogonal reaction, multi-component reaction, and metal chelate reaction. Among them, isocyanide-tetrazine reaction is a well-studied biomolecular labeling technology which enriches the potency of isocyanide as a bio-orthogonal handle. In addition, the methods of introducing isocyano groups into biomacromolecules are discussed in detail to facilitate the development of biomolecular markers in the future.

Choe et al. have described about the biodegradable plastics and the factors affecting the biodegradation of these plastics. The biodegradation levels reported by various groups or studies in different environments vary to a great extent. This article clarifies the common misconceptions and truths about biodegradation by bridging the three gaps in biodegradable plastics. These enable readers to accurately understand the current status and future development direction of real biodegradable plastics.

Xiao et al. established a high-throughput ICP-MS method for the detection of 62 elemental impurities in high-matrix calcium carbonate. The impurity content in calcium carbonate preparation from 9 manufacturers and two types of raw materials (light calcium carbonate and ground calcium carbonate) was examined, which showed that ground calcium carbonate was more suitable for the pediatric supplements.

Wang et al. discussed the structure and physiological functions of mitochondria and mitochondria dysfunction related diseases. Moreover, the research progress of mitochondria-targeting agents based on small molecules, biomolecules, and nanomaterial and their potential in targeted drug delivery, novel biological applications and therapeutic strategies is comprehensively described. Therefore, this study will greatly promote the design and exploration of mitochondrial targeting molecules and their targeting mechanisms.

Fasfous et al. prepared Fe/Ti-oxide modified carbon nanotube nanocomposites and analyzed its ability to adsorb Co ions in a simulated solution. The presence of CNTs hampers the growth of Fe_3_O_4_ and TiO_2_ particles and forming smaller nano-particles leading to better Co removal from solution. Better Co retention was observed at higher Ti/Fe loads and lower mass of CNTs. The results show that the combination of Fe/Ti oxide and CNTs has a synergistic effect on the retention of cobalt ions.

Yasmeen et al. were inspired by the use of graphics to manipulate chemical structures and nanostructures and studied the largest-edge Mostar invariant of an n-vertex cactus graph with a fixed number of cycles. Some lemmas have been proved by using graph transformations and calculations.

Wu et al. designed and synthesized a series of pyrimidine derivatives containing amide groups. These synthetic pyrimidine derivatives were verified for their biological activity through *in vitro* antifungal experiments. The results show that 5-Bromo-2-fluoro-N-(3-((2-methyl-6-(trifluoromethyl)Pyrimidin-4-yl)oxy)phenyl)benzamide has excellent antifungal activity, which also shows that the compound has the potential to be developed as a pesticide.

We have presented here some snippets of different research activities in the field of chemical biology. We expect that this will be a handy reference tool for students and researchers working in different areas of chemistry to carry out biological studies of some novel systems having potential applications.

